# Semaphorin 3A Suppresses Tumor Growth and Metastasis in Mice Melanoma Model

**DOI:** 10.1371/journal.pone.0033633

**Published:** 2012-03-20

**Authors:** Goutam Chakraborty, Santosh Kumar, Rosalin Mishra, Tushar V. Patil, Gopal C. Kundu

**Affiliations:** 1 National Center for Cell Science (NCCS), NCCS Complex, Pune, India; 2 Department of Histopathology, YCM Hospital, Pune, India; Enzo Life Sciences, Inc., United States of America

## Abstract

**Background:**

Recent understanding on cancer therapy indicated that targeting metastatic signature or angiogenic switch could be a promising and rational approach to combat cancer. Advancement in cancer research has demonstrated the potential role of various tumor suppressor proteins in inhibition of cancer progression. Current studies have shown that axonal sprouting inhibitor, semaphorin 3A (Sema 3A) acts as a potent suppressor of tumor angiogenesis in various cancer models. However, the function of Sema 3A in regulation of melanoma progression is not well studied, and yet to be the subject of intense investigation.

**Methodology/Principal Findings:**

In this study, using multiple *in vitro* and *in vivo* approaches we have demonstrated that Sema 3A acts as a potent tumor suppressor *in vitro* and *in vivo* mice (C57BL/6) models. Mouse melanoma (B16F10) cells overexpressed with Sema 3A resulted in significant inhibition of cell motility, invasiveness and proliferation as well as suppression of *in vivo* tumor growth, angiogenesis and metastasis in mice models. Moreover, we have observed that Sema 3A overexpressed melanoma clone showed increased sensitivity towards curcumin and Dacarbazine, anti-cancer agents.

**Conclusions:**

Our results demonstrate, at least in part, the functional approach underlying Sema 3A mediated inhibition of tumorigenesis and angiogenesis and a clear understanding of such a process may facilitate the development of novel therapeutic strategy for the treatment of cancer.

## Introduction

Melanoma or malignancies of melanocytic tissues have been identified as one of the most malignant cancer in the United States and around the world. In the year 2010, more than 68,130 new cases of melanoma have been reported in the United States with a result of 8,700 deaths [1]. Malignant progression of cancer cells depends on intrinsic crosstalk between several factors, overexpression of various oncogenic molecules and loss of function of tumor suppressor genes. Therefore, understanding the mechanisms of various tumor suppressor genes in regulation of cancer progression and their possible role in cancer therapeutics is under intense investigation. Semaphorins have been originally known as a large family of evolutionary conserved axonal guidance molecules [2], [3]. The role of semaphorins in various physiological as well as pathophysiological processes including cell migration, regulation of immune response, angiogenesis and cancer have recently been studied [4]. Among various semaphorins, selected members of semaphorin 3 (Sema 3) family are involved in suppression of tumor progression and have been considered as potent tumor suppressors [5]. Loss of expressions of Sema 3B and Sema 3F gene (deletion of chromosome 3p21.3 in human) have been shown to associate with lung cancer progression [6]–[8]. On the other hand, overexpression of these molecules inhibits tumor cell proliferation and *in vivo* tumor growth [9]–[12]. Moreover, Semaphorin 3A (Sema 3A), another member of this family is shown to inhibit angiogenesis and acts as tumor suppressor [13]–[16].

Sema 3A is originally described as a secretory protein with potent axonal repulsive activity [17], [18]. Polleux et al have identified the chemoattractive effect of Sema 3A on cortical apical dendrites [19] and shown that Sema 3A acts as a crucial regulatory molecule for neuronal development. However, Serini et al have observed a significant vascular defect in Sema 3A null mice [16]. In this study, we have deciphered the function of Sema 3A beyond brain, and demonstrated that this protein could play an important role in melanoma growth. Knockdown of endogenous Sema 3A significantly induce *in vitro* migration of human breast cancer cell and indicated that Sema 3A may act as a potent tumor suppressor [20]. Overexpression of Sema 3A attenuates invasion and matrigel adhesion of human prostate cancer cells [21]. Moreover, loss of Sema 3A inhibitory loop in hormone-refractory human prostatic cancer has been recently identified by tissue microarray analysis and further suggested that deregulation of Sema 3A pathway could be an important therapeutic target for prostate cancer progression [22]. Furthermore, overexpression of Sema 3A significantly suppresses *in vivo* breast tumor growth in mouse xenograft model [23]. However, the function of Sema 3A in regulation of melanoma progression is not well studied, and yet to be a field of intense investigation.

Angiogenesis, or formation of new blood vessel from the existing one has been considered as the most important step during tumor progression [24]. Current advancement in cancer research has shown that targeting angiogenic pathways could be a more rational and promising anti-cancer therapeutic approach [25]. To date, vascular endothelial growth factor (VEGF) is considered as one of the most potent angiogenic factor that governs tumor angiogenesis [26]. Interaction between VEGF and one of its co-receptor neuropilin 1 (NRP1) is known to play an important role in tumor angiogenesis [27], [28] and therefore blocking their interaction could be a rational anti-angiogenic therapeutic approach for cancer treatment [28], [29]. Moreover, NRP1 has been identified as one of the co-receptors of Sema 3A [30], [31]. Miao et al. have shown that Sema 3A and/or VEGF act as competitive ligand for binding to NRP1 and described that Sema 3A can attenuate VEGF-induced endothelial cell motility [32]. Thus, Sema 3A could act as a potent inhibitor of tumor angiogenesis by disrupting the interaction between VEGF and NRP1. Very recently, using transgenic mice model, Maione et al. have shown that Sema 3A acts as an endogenous angiogenesis inhibitor that blocks tumor growth by normalizing tumor vasculature [33]. Earlier data have shown that Sema 3F, another member of Sema 3 family, attenuates melanoma progression by inhibiting angiogenesis [34]. However, the correlation between Sema 3A expression and melanoma progression as well as angiogenesis is not clearly understood. Therefore, using multiple models, we have demonstrated the novel function of Sema 3A in suppression of melanoma progression and angiogenesis. Our study might be useful to develop more rational Sema 3A-mediated therapeutic strategy for the next generation of cancer management.

## Materials and Methods

### Cell lines, reagents and antibodies

The murine melanoma cell lines (B16F1 and B16F10) and human melanoma cell lines (A375 and SK-Mel-28) were purchased from American Type Culture Collection (ATCC, Manassas, VA), cultured in DMEM supplemented with 10% fetal bovine serum, 100 units/ml penicillin, 100 mg/ml streptomycin and 2 mM glutamine in a humidified atmosphere of 5% CO2 and 95% air at 37°C. Human umbilical vein endothelial cells (HUVEC) were cultured in EBM according to the manufacturer's instructions (Lonza, Walkersville, MD). Human recombinant Sema 3A (Cat no. AF1250-S3-025) and mouse recombinant Sema 3A (Cat no. 5926-S3-025) were purchased from R&D Systems. Goat anti-Sema 3A (Cat no. SC 1148), rabbit anti-α-tubulin (SC-135659), rabbit anti-PARP (SC-25780) antibodies were purchased from Santa Cruz Biotechnology (Santa Cruz, CA). Rabbit anti-vWF antibody (Cat no. HPA002082), PI, DAPI, FITC-conjugated phalloidin, fibronectin, Dacarbazine (DTIC), curcumin were purchased from Sigma (St. Louis, MO). Boyden type cell migration chambers were obtained from Corning (Corning, NY). Growth factor depleted matrigel and matrigel coated invasion chambers were obtained from BD Bioscience (Bedford, MA). Mouse anti-Sema 3A (Cat no. MAB-1250) and anti-neuropilin1 (Cat no. AF566) antibodies were purchased from R&D System (Minneapolis, MN). Rabbit anti-phospho p53 (Ser-15) (Cat no. 9284) antibody was obtained from Cell Signaling Technologies (Danvers, MA). The C57BL/6 mice were maintained at Experimental Animal Facility (EAF) of National Center for Cell Science (NCCS), Pune, India. The study was approved by Institutional Animal Care and Use Committee (IACUC), NCCS (Project Approval No. NCCS/EAF/2007/B116). Sema 3A siRNA was purchased from Dharmacon International (Lafayette, CO). Human Sema 3A and GAPDH specific TaqMan gene expression quantitative real time PCR (Q-PCR) assay related reagents were procured from Applied Biosystems (Carlsbad, CA). BrdU labeling and detection kit was purchased from Roche. Anti-Goat Cy2 and anti-rabbit Cy3 were purchased from Chemicon International.

### RNA isolation, semi-quantitive RT–PCR and Q-PCR

RNA isolation and reverse transcription-polymerase chain reaction (PCR) were performed as described earlier [35]. Total RNA was isolated from B16F1, B16F10, A375 and SK-Mel-28 cells by using Trizol reagent (Invitrogen) and reverse transcription-PCR was performed using following sets of primers: Sema 3A forward: 5′-CAG CCA TGT ACA ACC CAG TG-3′; Sema 3A reverse: 5′-ACG GTT CCA ACA TCT GTT CC-3′; GAPDH forward: 5′-ACT CCA CTC ACG GCA AAT TC-3′; GAPDH reverse: 5′-CCT TCC ACA ATG CCA AAG TT-3′. Q-PCR was performed with TaqMan gene expression assay (Applied Biosystems) according to manufacture's instruction. GAPDH was used as internal control. The PCR products were analyzed by electrophoresis using 1.0% agarose gel.

### Generation of Sema 3A stable clones

Human Sema 3A cDNA in PCEP4 expression vector (H-Sema 3A-AP; a generous gift from Prof. Alex Kolodkin, Johns Hopkins University School of Medicine, Baltimore, Maryland, USA) was transfected in B16F10 cells using lipofectamine 2000 (Invitrogen; Carlsbad, CA). After transfection, cells were selected with 400 µg/ml hygromycin and three positive clones were isolated and denoted as clone 1, 2 and 3.

### Melanoma clinical specimen analysis

Normal skin biopsy and malignant melanoma specimens were collected from local hospitals with informed consent. Tissue sections were stained with hematoxylin and eosine and the occurrence of melanoma in these samples was analyzed with the help of expert onco-pathologist. The expression profiles of Sema 3A and phospho p53 (Ser-15) were analyzed by immunofluorescence in tissue sections using their specific antibodies and visualized under confocal microscopy (Zeiss) as described earlier [35], [36].

### Western Blot Analysis

The expression of Sema 3A in control or Sema 3A clones was determined by Western blot using anti-Sema 3A antibody as described [28]. The effect of Sema 3A on curcumin-induced PARP cleavage was also determined using anti-PARP antibody.

### Matrigel colony formation assay

Matrigel colony formation assay was performed on growth factor depleted matrigel coated plate. Briefly, 250 µl cold matrigel was coated on 24 well plates and the plates were kept at 37°C for 1 h for polymerization. Equal number of cells (control B16F10 or clone 2) were grown on matrigel coated plates and incubated at 37°C for 7 days. After termination of experiments, colonies were visualized under microscope (Nikon) and photographed.

### Immunofluorescence study

Cells (control and clone 2) were grown on fibronectin coated cover slips for 6 h. After 6 h, cells were fixed with 2% paraformaldehyde and stained with FITC-conjugated phalloidin and visualized and photographed under fluorescence microscope (Zeiss).

In separate experiments, B16F10, clone2 and SK-Mel-28 cells were plated on cover slips and incubated in serum free media for 24 h. SK-Mel-28 cells were either treated with Sema-3A recombinant protein (100 ng/ml) or vehicle for 24 h. Cells were fixed with 2% paraformaldehyde, stained with anti-Ser15-phospho-p53 antibody and visualized under confocal microscope (Zeiss). Nuclei were stained with PI. To analyze the effect of curcumin on nuclear morphology, equal number of cells (control B16F10 and clone 2) were plated on cover slips and treated with indicated concentrations of curcumin. Cells were fixed and stained with propidium iodide as described earlier and visualized under fluorescence microscope [37].

### Co-migration and co-invasion assays

Invasion assay was performed in matrigel coated invasion chambers (BD Bioscience) using control B16F10 and clone 2 cells as described [38]. Briefly, B16F1, B16F10 or clone 2 were added to the upper portion of the Boyden chamber and incubated at 37°C for 18 h. In separate experiments, conditioned media collected either from clone 2 (Sema 3A overexpressed) or Sema 3A siRNA transfected B16F1 or B16F1 (with or without Sema 3A antibody) were used in the lower chamber as chemoattractant for B16F10 migration. In another experiments, A375 and SK-Mel-28 cells, treated with 100 ng/ml of Sema 3A were used in the upper portion of invasion chambers. FBS was used as chemoattractant. The cells invaded to the reverse side of the filter were stained, photographed, counted in 3 high-power fields (C/HPF) under an inverted microscope (Nikon), analyzed statistically and represented in the form of bar graph.

The endothelial-melanoma cell interaction was shown by direct co-migration or co-invasion assays as described [28]. Briefly, HUVEC were added in upper chamber and control B16F10 or clones 2 cells were used in lower chamber. Anti-NRP1 blocking antibody was used in upper chamber. In separate experiments, conditioned media collected either from clone 2 (Sema 3A overexpressed) or Sema 3A siRNA transfected B16F1 or B16F1 (with or without Sema 3A Ab) were used in the lower chamber. FBS serve as chemoattractant for HUVEC migration and invasion. After 18 h, the migrated or invaded HUVEC in the reverse side of the filter were stained, photographed, analyzed statistically and represented graphically.

### BrdU incorporation assay

SK-Mel-28 cells were seeded on coverslip and treated with 100 ng/ml Sema 3A for 24 h followed by incubation with complete media supplemented with BrdU for another 24 h. Cells were fixed with 100% cold methanol and stained with BrdU labeling and detection kit (Roche), visualized under fluorescence microscope, photographed and analyzed.

### Wound assay and time laps microscopy

To further confirm the motility of control B16F10 and clone 2 cells, wound assay was performed as described [28], [36]. Briefly, clone 2 cells either alone or treated with anti-Sema 3A or anti-NRP1 blocking antibody were used for wound assay. In separate experiments, control B16F10 cells either alone or treated with conditioned media obtained from clone 2 were used. After 18 h, wound photographs were taken under inverted microscope (Nikon). The wound migration was also visualized under time laps microscopy. Confluent monolayer of cells was wounded and the migration of the cells towards the wound was monitored and photographed under Nikon time laps microscope in an interval of 10 min up to 18 h and represented in the form of video using Image Pro-Plus software after compiling the images into a movie. To further validate the effect of endogenous Sema 3A on melanoma migration, B16F1 cells were either silenced using Sema 3A specific siRNA or pretreated with Sema 3A blocking antibody and analyzed for wound assay. Migration assay was also performed using A375 and SK-Mel-28 cells treated with Sema 3A (100 ng/ml) for 18 h.

### MTT assay

The effect of Sema 3A on melanoma cell proliferation was determined by MTT (3-{4,5-dimethylthiazol-2-yl}-2,5-diphenyl tetrazolium bromide) assay. Briefly, control B16F10 or clone 2 cells were plated and allowed to grow at 37°C. Both cells were treated with Dacarbazine (0–400 µM) and curcumin ((0–50 µM) and MTT was added to the cells and incubated at 37°C for 4 h. The isopropanol was added to the cells, absorbance was measured in an ELISA reader (Bio-Rad) at 570 nM.

### DNA ladder assay

Both control B16F10 and clone 2 cells either untreated or treated with two different concentrations of curcumin were incubated at 37°C for 12 h, lysed in ladder assay buffer (10 mM Tris-HCl, pH 8.0 containing 1 mM EDTA, 0.1% SDS and 50 mg/ml Proteinase K) and genomic DNA was isolated by isopropanol precipitation method. Equal amount of DNA was analyzed by 2% agarose gel electrophoresis.

### *In vivo* tumorigenicity, *in vivo* invasion, histopathology and immunohistochemistry

Control B16F10 and clone 2 cells (1×10^6^) were injected subcutaneously into the dorsal flank region of C57BL/6 male mice (6–8 weeks old). In separate experiments, serum free conditioned media collected from clone 2 cells was injected twice a week into the tumor site of mice generated by control cells. Tumor length and width were measured every week. After 4 weeks, mice were sacrificed, photographed, tumors were dissected out, weighted and used for histopathological and immunohistochemical studies. The mice were also dissected ventrally, photographed and the metastasized organs such as liver, intestine and kidney were used for histopathological studies.

### *In vivo* metastasis study by intra-cardiac injection

Control B16F10 and clone 2 cells (1×10^5^) were injected intracardiacly into anesthetized C57BL/6 male mice (6–8 weeks old). After 3 weeks, mice were sacrificed; photographed, metastasized organs (lung, liver and intestine) were dissected out and used for histopathological studies.

### Statistical analysis

The cell migration, invasion, MTT assays, tumor-endothelial interaction assays, tumor weights and volumes were analyzed statistically. Statistical differences were determined by paired Student's t test. Differences were considered significant when P<0.05.

## Results

### Expression profile of Sema 3A in human melanoma clinical specimens and its correlation with melanoma growth

**Figure 1:**
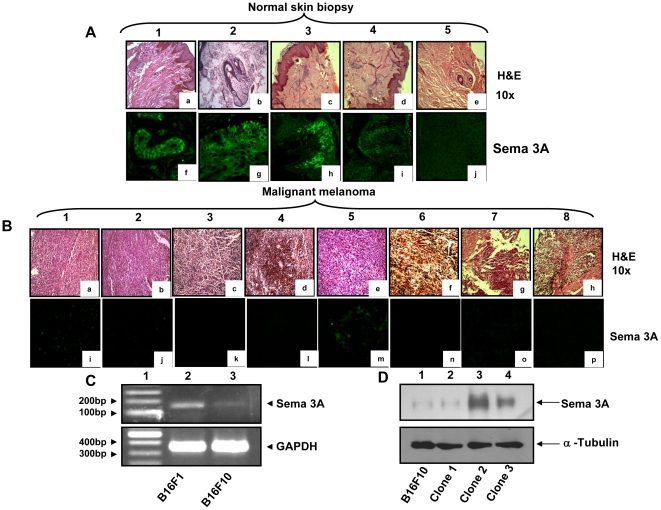
Histopathological and immunohistochemical analyses of human normal skin biopsy and malignant melanoma tissues. (**A&B**) Five normal skin biopsy and eight malignant melanoma specimens were stained with hematoxylin and eosine and the histopathologic micrographs were visualized at 10× magnifications (Fig. 1A, panels a–e, & Fig. 1B, panels a–h). Tissue sections were also analyzed immunohistochemially for visualizing the expression of Sema 3A (Fig. 1A, panels f–j & Fig. 1B, panels i–p). Sema 3A was stained with Cy2 (Green). Note that significant loss of Sema 3A expression was detected in malignant melanoma tissue samples as compared to normal skin samples. (**C**) Endogenous expression of Sema 3A mRNA in B16F1 and B16F10 cells as detected by RT-PCR. GAPDH was used as loading control. (**D**) Expression of Sema 3A in B16F10 clones were analyzed by Western blot using specific antibody. The second clone (lane 3, denoted as clone 2) shows significantly higher expression of Sema 3A as compared to other clones and control B16F10 cells. The data represent three independent experiments exhibiting similar results.

Clinicopathological studies on human melanoma specimens have shown that melanoma progression is associated with angiogenesis [39]. In this study, we have analyzed 8 human melanoma tissue samples and 5 normal skin biopsy samples by histopathology and immunohistochemistry. The clinical samples were collected from local hospital with informed consent. The occurrences of melanoma in these samples were examined by H&E staining and the micrographs were taken in 10× magnification (Fig. 1A, panel a–e and 1B panel a–h). The expression level of Sema 3A in these samples was determined by immunohistochemistry using anti-Sema 3A antibody. The results revealed that significant expression of Sema 3A was observed in normal skin biopsy specimens (Fig. 1A, panel f–j). However, the level of Sema 3A was significantly reduced in all 8 melanoma tissue sections indicating that loss of Sema 3A level may be linked with melanoma growth (Fig. 1B, panel i–p). Taken together, these data suggested that loss of Sema 3A expression is associated with melanoma progression in human clinical specimens.

### Generation of Sema 3A clone in murine melanoma cells

To examine the endogenous level of Sema 3A, we have used two different murine melanoma cell lines. Our RT-PCR and Western blot analysis data revealed that low metastatic melanoma cells (B16F1) express significantly higher level of Sema 3A as compared to highly metastatic (B16F10) cells (Fig. 1C and data not shown). To further study the level of Sema 3A in human melanoma cells (A375 and SK-Mel-28), Q-PCR was performed. The data showed that A375 cells have significantly higher Sema 3A expression than SK-Mel-28 cells (Fig. S1A).

To investigate the role of Sema 3A on melanoma progression, we generated Sema 3A positive stable clone in highly metastatic B16F10 cells as described under material and methods. Our results revealed that among three hygromycin resistance Sema 3A clones, clone 2 expressed high level of Sema 3A as compared to clones 1 and 3 (Fig. 1D). Hence clone 2 is used for all further studies.

### Overexpression of Sema 3A suppresses metastatic phenotype of B16F10 cells

**Figure 2:**
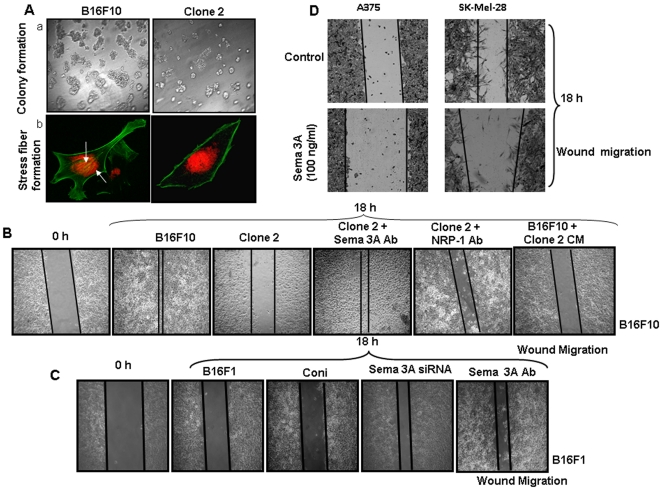
Loss-and-gain of Sema 3A functions and it's correlation with metastatic phenotype of melanoma cells. (**A**) panel a, equal number of control B16F10 and clone 2 cells (1×10^4^) were grown on matrigel coated plate and incubated at 37°C for 7 days. Colony photographs were taken at 10× magnification. Panel b, control B16F10 and clone 2 cells (1×10^4^) were plated on fibronectin coated coverslips and incubated for 6 h. Cells were fixed in 2% PFA and stained with FITC-conjugated phalloidin (green). Stress fibers were shown by arrows. Nuclei were stained with PI (red). Photographs were taken at 60× magnification. (**B**) The effect of Sema 3A on B16F10 cell motility was performed by wound migration assay. Control B16F10 or clone 2 positive cells were used for wound migration. In separate experiments, clone 2 positive cells either treated with anti-Sema 3A or anti-NPR1 blocking antibody were used for wound migration assay. In another experiments, control B16F10 cells were incubated with conditioned medium (CM) collected from clone 2 and wound migration assay was performed. After 18 h, cells were visualized and photographed under Nikon microscope at 10× magnification. (**C**) To further study the loss-of-function of Sema 3A in regulating melanoma cell migration, wound assay was performed using B16F1 cells. Cells were either transfected with Sema 3A siRNA or treated with Sema 3A blocking antibody and used for migration assay. (**D**) To study the gain-of-function, wound migration assays were performed using A375 or SK-Mel-28 cells treated with recombinant exogenous Sema 3A for 18 h. Sema 3A significantly attenuates wound motility of melanoma cells. All the data shown here are the representatives of triplicate independent experiments.

Earlier studies have shown that invasive behavior of melanoma cells is one of the key phenomena during melanoma progression [40]. Colony formation on matrigel has been frequently employed as a reliable assay to determine the *in vitro* tumorigenicity and metastatic phenotype of cancer cells [41], [42]. To study the effect of Sema 3A on *in vitro* tumorigenicity of melanoma cells, matrigel colony formation assay was performed. The results revealed that clone 2 cells exhibits significantly reduced colony formation on matrigel as compared to control B16F10 cells (Fig. 2A, panel a). To investigate the role of Sema 3A on stress fibre formation, both the control B16F10 and clone 2 cells were grown in fibronectin coated plates and stained with FITC-conjugated phalloidin. The results showed that there was significant increase in actin stress fiber (indicated by arrow) and lamellipodia formation in control cells as compared to clone 2 (Fig. 2A, panel b). These data suggested that overexpression of Sema 3A attenuates *in vitro* metastatic phenotype of melanoma cells.

### Effect of exogenous Sema 3A on melanoma cell migration and invasion

**Figure 3:**
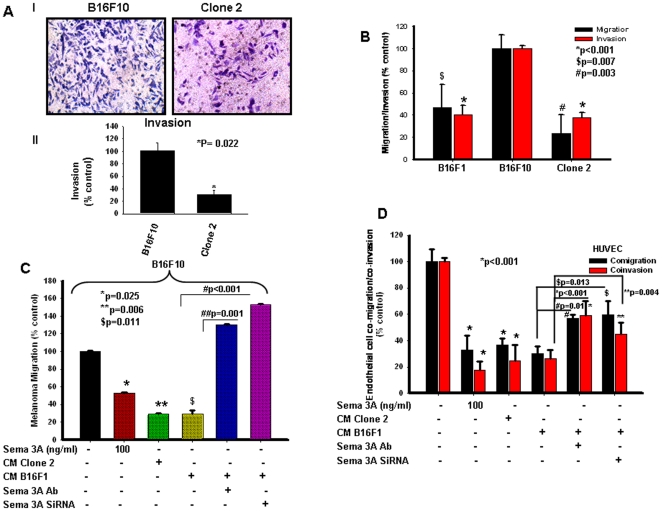
Sema 3A inhibits melanoma cell migration and invasion through autocrine and paracrine mechanisms. (**A**) Invasion assays were performed on matrigel coated invasion chamber. Cells (control or clone 2; 1×10^5^) were plated on upper chamber. After 18 h, invaded cells were stained with Giemsa and photographed (panel I), counted in 3 high-power fields (C/HPF) under an inverted microscope (Nikon), analyzed statistically and represented in the form of bar graph (panel II, *P = 0.022). (**B**) Autocrine mechanism of Sema 3A action on melanoma motility and invasiveness were demonstrated by migration and invasion assay using either B16F10 or clone 2 or B16F1 cells. *p<0.001, $p = 0.007, #p = 0.003 vs. B16F10. (**C**) Boyden chamber migration assay was performed using B16F10 cells to demonstrate the paracrine mechanism of action of Sema 3A. Conditioned media (CM) collected from clone 2 or B16F1 cells alone or B16F1 transfected with Sema 3A siRNA were used in the lower chamber as a chemoattractant. In separate experiments, either B16F10 cells treated with Sema 3A (100 ng/ml) or CM from B16F1 treated with Sema 3A antibody were used in the lower chamber. Migrated cells were counted, analyzed statistically and represented graphically. *p = 0.025, **p = 0.006 and $p = 0.011 vs. control, #p<0.001, ##p = 0.001. (**D**) Tumor-endothelial interaction was performed in modified Boyden chamber or matrigel coated invasion chamber. Endothelial cells were placed on upper chamber whereas similar conditioned media (as of Fig. 3C) were used in the lower chamber. Migrated or invaded cells were stained with Giemsa, photographed, analyzed statistically and represented in the form of graph. *p<0.001 vs. control, **p = 0.004, #p = 0.01, $p = 0.013.

To determine the effect of exogenous Sema 3A on human melanoma cell migration and invasion, we have used two different human malignant melanoma cell lines, A375 and SK-Mel-28. Both these cells exhibited reduced migration when treated with recombinant Sema 3A (Fig. 2D). We have also observed that untreated SK-Mel-28 cells exhibit significantly higher invasion as compared to A375 cells, however, treatment of exogenous Sema-3A significantly suppressed invasion in SK-Mel-28 as well as A375 cells (Fig. S1C). Taken together, these data showed that exogenous Sema 3A inhibits migration and invasiveness of human malignant melanoma cells. Moreover, we have observed drastic reduction of invasion of Sema 3A overexpressed melanoma cells (clone 2) as compared to control (Fig. 3A, panel I). The data were quantified and represented in the form of bar graph (Fig. 3A, panel II).

### Sema 3A abridged *in vitro* melanoma cell motility through autocrine and paracrine manner

To determine the effect of Sema 3A on melanoma cell motility, wound assay was performed as described [28]. The data indicated that overexpression of Sema 3A significantly attenuated *in vitro* melanoma cell motility (Fig. 2B). However, treatment of clone 2 with anti-Sema 3A or anti-NRP1 blocking antibody drastically induced cell migration as compared to clone 2-derived cells alone demonstrating that tumor derived Sema 3A inhibits tumor cell motility through NRP1 dependent autocrine manner (Fig. 2B). Conditioned media collected from clone 2 significantly suppressed wound migration of control B16F10 cells (Fig. 2B) further suggesting that tumor derived Sema 3A could also suppress tumor cell motility via paracrine mechanism. On the other hand, silencing endogenous Sema 3A or blocking Sema 3A activity in B16F1 cells showed enhanced cell migration (Fig. 2C). These data further demonstrated the significance of loss-of-function of Sema 3A in melanoma cell migration.

To further validate the suppressive effect of Sema 3A on real time melanoma cell motility, wound migration assay was performed using Time lapse microscopy under the same experimental conditions as described above. The video demonstrated that control B16F10 (18 h; Fig. S5) cells exhibit faster movement and complete closer of wound as compared to clone 2 cells (18 h; Fig. S6). Moreover, incubation of control B16F10 cells with conditioned media of clone 2 showed significant reduction of migration as well as exhibit similar motility phenotype like clone 2 cells (18 h; Fig. S7). These data further corroborate that clone 2 derived Sema 3A attenuates motility of control B16F10 cells. Taken together, the time lapse experimental data demonstrated that Sema 3A through an autocrine and/or paracrine manner inhibits melanoma cell motility and may act as potential suppressor of melanoma progression. To further demonstrate the role of Sema 3A in melanoma cell migration through autocrine and paracrine mechanism, Boyden chamber migration assay was performed where conditioned media (CM) collected from clone 2 or B16F1 cells (with or without Sema 3A siRNA transfection) were used in the lower chamber as chemoattractant. Moreover, B16F10 cells either treated with Sema 3A (100 ng/ml) or CM collected from B16F1 treated with Sema 3A antibody were also used in the migration assay. The data revealed that supplying exogenous Sema 3A can impede and silencing or inhibiting its activity can enhance B16F10 migration (Fig. 3C and Fig. S2A). These results demonstrated that Sema 3A regulates melanoma cell migration through autocrine (Fig. 3B and Fig. S1B) and paracrine (Fig. 3C and Fig. S2A) mechanism.

### Sema 3A attenuates melanoma cell proliferation

**Figure 4:**
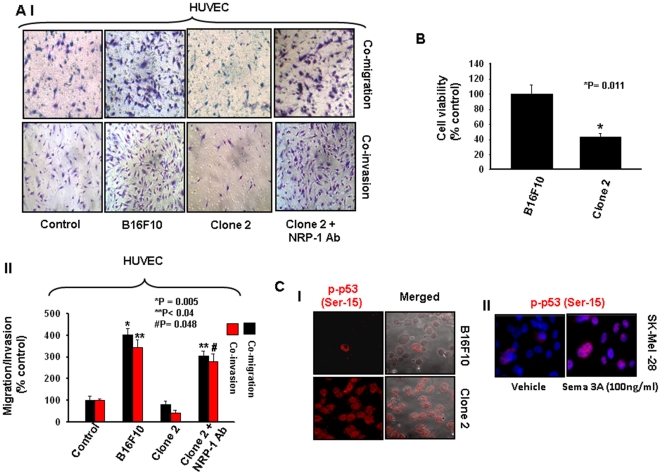
Sema 3A augments p53 phosphorylation and attenuates tumor-endothelial interaction via neuropilin 1 (NRP1) mediated paracrine mechanism. (**A**) Tumor-endothelial interaction was performed in modified Boyden chamber or matrigel coated invasion chamber. Endothelial cells (HUVEC) were placed on upper chamber whereas tumor (control B16F10 or clone 2) cells were used in lower chamber. In separate experiments, HUVEC were treated with anti-NRP1 blocking antibody and used on the upper chamber. After 18 h, migrated or invaded cells were stained with Giemsa, photographed (Fig. 4A, panel 1), counted in three high power field (C/HPF) and represented in the form of bar graph (panel II, *p = 0.005 vs. control, **p<0.04 vs. control, #p = 0.048 vs. control). The experiments were performed in triplicate. (**B**) Overexpression of Sema 3A inhibits cell viability of B16F10 cells. Equal number of control B16F10 or clone 2 cells (1×10^4^) were grown in 96 well tissue culture plates and cell viability was performed by MTT assay. The data are represented in the form of bar graph (*p = 0.011) and the mean value of triplicate experiments is indicated. (**C**) Sema 3A augments Ser-15 phosphorylation of p53. The phosphorylation at Ser-15 of p53 was analyzed by immunofluorescence using specific antibody followed by Cy3 (red) labeled secondary antibody. Photographs were taken under confocal microscope at 60× magnification (Fig. 4C, panel I). The typical photographs have shown here represents three independent experiments exhibiting similar results. Similarly, SK-Mel-28 cells were treated with Sema 3A (100 ng/ml) and the level of phospho-p53 at Ser-15 was analyzed by confocal microscopy (Fig. 4C, panel II).

To determine whether overexpression of Sema 3A exerts any effect on melanoma cell proliferation, MTT assay was performed. Equal number of control B16F10 and clone 2 cells were grown in serum free media for 24 h and then incubated with 0.5 mg/ml of MTT. The proliferation rate of control and clone 2 cells were analyzed by ELISA reader and plotted graphically (Fig. 4B). The data showed that overexpression of Sema 3A reduces the cell viability to 43% of the control. To further confirm this study, BrdU incorporation assay was performed using Sema 3A treated SK-Mel-28 cells. Cells were stained with BrdU labeling and detection kit, visualized under fluorescence microscope, photographed, analyzed and represented in the form of bar graph. The data showed significant reduction in BrdU incorporation in Sema 3A treated cells (Fig. S4 A and B).

### Enhanced expression of Sema 3A augments p53 phosphorylation

p53, a tumor suppressor protein plays crucial role in regression of cancer progression [43]. Recent studies have revealed that phosphorylation of Ser-15 residues of p53 exhibit growth retardation in melanoma [44]. Tedeschi et al reported that growth cone retraction by Sema 3A is overcomed by cGMP in wild type but not in p53 null dorsal root ganglia [45]. In this study, we have observed that overexpression of Sema 3A inhibits *in vitro* tumorigenic phenotype of melanoma cells. Therefore, we sought to determine whether Sema 3A has any role in suppression of melanoma progression and the involvement of activated p53 in this process. Accordingly, control and clone 2 cells were analyzed by immunofluorescence using anti-phospho p53 (Ser-15) antibody and analyzed by confocal microscopy (Zeiss). The results indicated that cells overexpressing Sema 3A enhances p53 phosphorylation at Ser-15 residue (Fig. 4C, panel I) suggesting the possible involvement of activated p53 in Sema 3A regulated melanoma progression. To further validate our findings, SK-Mel-28 cells were treated with Sema protein (100 ng/ml) for 60 min and analyzed by immunofluorescence using anti-phospho p53 (Ser-15) antibody (Fig. 4C, panel II). The results indicated that exogenous supply of Sema 3A enhances p53 phosphorylation at Ser-15 residue. To further correlate p53 and Sema 3A in clinical samples, we analyzed the expression profile of Sema 3A and phospho-p53 in normal as well as malignant melanoma clinical specimens. These data suggested the enhanced expression of Sema 3A and phospho-p53 in normal skin samples as compared to malignant melanoma specimens (Fig. S3A and B). These findings further strengthened the correlation between p53 and Sema 3A in melanoma progression.

### Tumor-derived Sema 3A attenuates melanoma-endothelial cell interaction through NRP1 dependent paracrine manner

Our earlier studies have demonstrated that tumor-endothelial interaction plays crucial role in tumor angiogenesis which ultimately promotes tumor progression and angiogenesis [28]. To study the role of Sema 3A in tumor-endothelial interaction through autocrine and paracrine mechanisms, co-migration and co-invasion assays were performed using HUVEC and melanoma cells. Conditioned media (CM) collected from clone 2 or B16F1 cells (with or without Sema 3A siRNA transfection) or treated with Sema 3A antibody were used in the lower chamber. Moreover, HUVECs were also treated with Sema 3A (100 ng/ml) and used in upper chamber for co-migration and co-invasion assays. The data depicted that providing exogenous Sema 3A can reduce and silencing or blocking endogenous Sema 3A can enhance HUVEC migration/invasion (Fig. 3D and Fig. S2B), suggesting that Sema 3A regulates melanoma-endothelial interactions through paracrine mechanism.

Moreover, we have observed the involvement of NRP1 in tumor-endothelial interaction [28]. NRP1 is identified as one of the cell surface receptor that interacts with Sema 3A [30], [31]. Therefore, to investigate the effect of Sema 3A on tumor-endothelial interaction, co-migration and co-invasion assays were performed as described in material and methods. Our results revealed that cells overexpressing Sema 3A (clone 2) exhibit reduced migration and invasion of HUVEC towards tumor cells (Fig. 4A, panels I and II). However, blocking the endothelial cell-derived NRP1 has reversed these effects (Fig. 4A, panels I and II). Taken together our results suggested that overexpression of Sema 3A regulates tumor-endothelial cell interaction through NRP1 dependent paracrine mechanism.

### Sema 3A sensitizes melanoma cells in response to various anti-cancer agents

**Figure 5:**
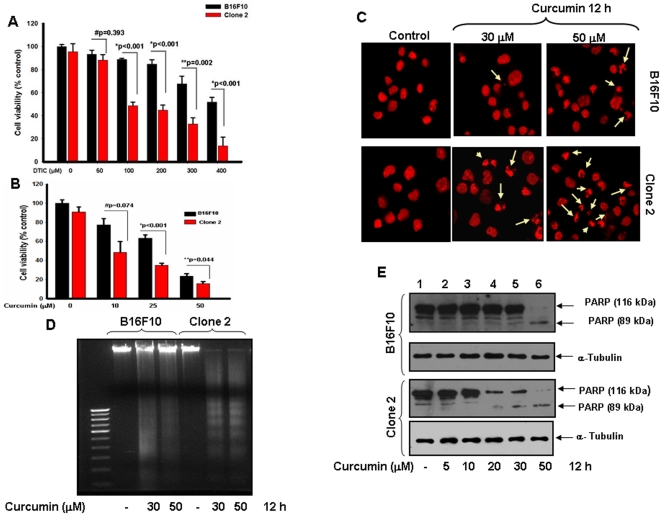
Sema 3A overexpressed clone exhibits increased drug sensitivity in B16F10 cells. (**A**) Control or clone 2 cells were treated with melanoma specific drug, Dacarbazine (DTIC) (0–400 µM) for 24 h and cells survival was analyzed by MTT assay. #p = 0.393, *p<0.001, **p = 0.002 vs. respective treatment group within B16F10 and clone 2 cells. (**B**) Similarly, both cells were treated with curcumin (0–50 µM) for 12 h and cell viability was checked by MTT assay. The data are represented in the form of bar graph and the mean value of triplicate experiments is indicated. #p = 0.074, *p<0.001, **p = 0.044 vs. respective treatment group within B16F10 and clone 2 cells. (**C**) Both cells were treated with indicated concentrations of curcumin, fixed and stained with PI (red) and photographed under fluorescence microscope at 60× magnifications. The apoptotic nuclei are indicated by arrows. (**D**) Cells were treated with two doses of curcumin for 12 h. Fragmentation of genomic DNA was extracted and resolved on 2% agarose gel. Apoptotic DNA fragmentation was visualized by ethidium bromide staining. (**E**) Curcumin induced apoptosis in control and clone 2 cells were also analyzed by Western blot using anti-PARP antibody. Cells were treated with 0–50 µM curcumin for 12 h and then analyzed by Western blot. α-Tubulin was used as loading control. The experiments showed here are representative of triplicate independent experiments with similar results.

To examine the effect of various anti-cancer agents in absence or presence of Sema 3A on melanoma cell death, cell viability assay was performed. Briefly, both control B16F10 and clone 2 cells were exposed with various anti-cancer agents like curcumin (0–50 µM) and Dacarbazine (0–400 µM) for 12 h and 24 h respectively, and the cell viability was determined by MTT assay. The results have shown that Sema 3A significantly sensitizes melanoma cells in response to these agents in a dose dependent manner (Fig. 5A & B).

### Curcumin selectively promotes apoptosis in response of Sema 3A

Earlier we and others have reported that curcumin with higher doses significantly reduced cell viability and induce apoptotic phenotype in B16F10 cells [38], [46]. In this study, we have observed that curcumin significantly suppresses the survival of Sema 3A overexpressing melanoma cells as compared to control B16F10 cells (Fig. 5B). To further elucidate the effect of curcumin on cell survival in presence of Sema 3A, both control and clone 2 cells were incubated with two doses of curcumin, fixed and nuclei were stained with propidium iodide (PI) and visualized under fluorescence microscope. The data showed that curcumin even in lower doses in clone 2 cells is able to induce apoptotic morphology as compared to parental cells (Fig. 5C).

To further validate the effect of curcumin on cell death, DNA fragmentation assay was performed. The data showed that clone 2 cells along with low doses of curcumin significantly induce apoptosis as characterized by marked DNA fragmentation (Fig. 5D). The curcumin-induced apoptosis was also analyzed by Western blot using anti-PARP antibody (Fig. 5E). The results also indicated that curcumin even in low dose is able to induce PARP cleavage in clone 2 cells.

### Sema 3A suppresses *in vivo* melanoma growth and angiogenesis in C57BL/6 allograft melanoma model

**Figure 6:**
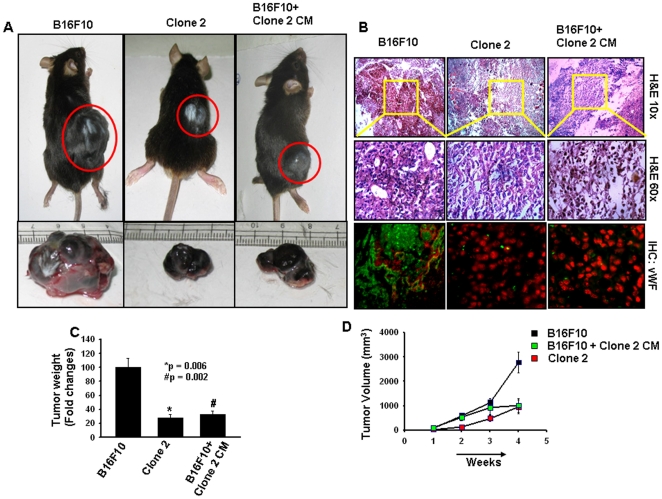
Overexpression of Sema 3A abrogates *in vivo* melanoma growth and angiogenesis in subcutaneous allograft tumor model in C57BL/6 mice. Control B16F10 and clone 2 cells (1×10^6^/mice) were injected subcutaneously into the dorsal flank region of male mice (6–8 weeks old; n = 6). In separate experiments, serum free conditioned media (CM) collected from clone 2 cells were injected intratumorally twice a week to the tumors generated by B16F10 cells upto termination of the experiments (n = 6). Mice were sacrificed after 4 weeks. (A) Typical photographs of subcutaneous melanoma in C57BL/6 mice and excised tumors of respective mice were shown. (B) Mice allograft tumors were analyzed by histopathology and immunohistochemistry using anti-vWF antibody. vWF was stained with Cy2 (green) whereas nuclei were countered stained with PI (red). (C) Weight of the excised tumors were measured, analyzed and represented in the form of bar graph (*p = 0.006, ^#^p = 0.002). (D) Tumor volumes of the allograft melanoma tumors were measured weekly, analyzed and plotted graphically. Six mice were used in each set of experiments.

Our *in vitro* experimental results prompted us to investigate the role of Sema 3A on *in vivo* melanoma progression. Accordingly, control B16F10 and clone 2 cells were injected subcutaneously to C57BL/6 mice (n = 6/group). In separate experiments, conditioned media (CM) collected from clone 2 cells were injected intratumorally, twice a week to the tumors generated by injecting B16F10 cells. After 4 weeks, mice were sacrificed and photographed, tumors were removed and weighed and represented in the form of bar graph (Fig. 6A and C). Tumor volumes were measured, analyzed and represented graphically (Fig. 6D). The data showed that overexpression of Sema 3A significantly suppressed *in vivo* tumor load in C57BL/6 mice. Moreover, intratumoral injection of CM from clone 2 attenuates tumor growth of control B16F10 cells in these mice (Fig. 6A and C) demonstrating the paracrine effect of secreted Sema 3A in regression of melanoma growth.

The tumor sections were analyzed histopathologically using H&E staining and the photographs were taken at 10× and 60× magnifications (Fig. 6B). The data showed that tumors generated by control B16F10 cells exhibit higher infiltration, poorly differentiated structure, enhanced nuclear polymorphism and increased number of tumor giant cells as compared to the tumors generated by clone 2 or CM of clone 2 (Fig. 6B). The data demonstrated that Sema 3A significantly attenuates *in vivo* melanoma growth. The tumor sections were also analyzed immunohistochemically using anti-vWF antibody. The results showed that there is a significant enhancement of tumor angiogenesis in tumor sections generated by control B16F10 cells as compared to clone 2 (Fig. 6B), indicating that overexpression of Sema 3A attenuates melanoma growth and angiogenesis in allograft tumor models via an autocrine or paracrine mechanism.

**Figure 7:**
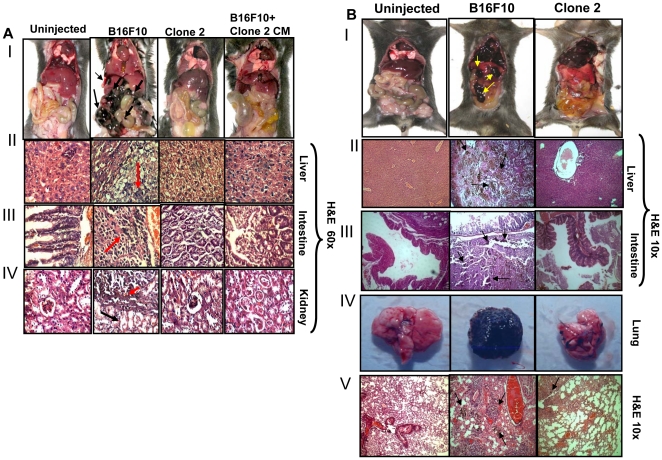
Sema 3A overexpression attenuates melanoma metastasis. (**A**) The mice described in Fig. 6A, were dissected ventrally and photographed. Growth of melanoma tumors were indicated by arrows (Fig. 7A, panel I). Internal organs (liver, intestine and kidney) were analyzed histopathologically and the micrographs were shown at 60× magnification (Fig. 7A, panels II–IV). Melanoma positive cells in metastatic lesions were marked by red arrows, whereas surrounding stromal regions were indicated by black arrows. Six mice were used in each group. Un-injected mice are used as control. (**B**) Sema 3A attenuates melanoma metastasis in mice. Control or clone 2 cells were injected intracardiaclly and mice were sacrificed, dissected ventrally and photographed (Fig. 7B, panel I). Metastasized organs were indicated by arrows. Liver and intestine of the mice were analyzed histopathologically by H&E staining (Fig. 7B, panels II & III). Micrographs were taken at 10× magnification. Arrows showed the melanoma foci. Fig. 7B, panel IV shows the excised lung of respective mice. Lung sections were also analyzed histopathologically and photographs were taken at 10× magnification (Fig. 7B, panel V). Melanoma metastatic foci in lung sections are indicated by arrows. Six mice were used in each set of experiment. Un-injected mice were used as control.

To further examine the potential role of Sema 3A in tissue specific metastasis in melanoma models, the allograft melanoma tumors as shown in Fig. 6A, were ventrally dissected and metastatic lesions such as liver, intestine and kidney were separated and analyzed by histopathologically (60×) (Fig. 7A, panels II–IV). The data indicated that tumors generated by control cells augment metastasis in liver, intestine and kidney whereas Sema 3A clone 2 or CM of clone 2 dramatically suppressed these metastasis (Fig. 7A, panels I–IV) demonstrating that Sema 3A inhibits melanoma growth, angiogenesis and metastasis in subcutaneous C57BL/6 mice models.

### Sema 3A attenuates melanoma metastasis

To determine whether Sema 3A inhibits melanoma metastasis, we implanted melanoma (control or clone 2) cells into the arterial circulation via intracardiac injection. After 18 days, mice were sacrificed, dissected and photographed (Fig. 7B, panel I). Mice with uninjected cells were used as control. The data suggested the significant melanoma metastasis in liver and intestine of control B16F10 but not in clone 2 injected mice (Fig. 7B, panels II & III). Moreover, we have detected several metastatic foci in the tissue sections of liver and intestine of control B16F10 injected mice by histopathology using H&E staining. The lungs of these mice were photographed and analyzed histopathologically. Significant lung metastasis was observed in control B16F10 but not in clone 2 cells injected mice (Fig. 7B, panels IV & V). Overall, the data clearly provided evidence that overexpression of Sema 3A significantly attenuates melanoma metastasis to lung, liver and intestine in murine melanoma model.

## Discussion

In this study, using various *in vitro* as well as *in vivo* models, we have demonstrated for the first time that Sema 3A could act as a potential tumor suppressor in melanoma model. The data also revealed that Sema 3A could be a rational and effective therapeutic molecule for treatment of melanoma. Our conclusion is supported by several lines of evidences: (i) expression profile of Sema 3A is negatively correlated with melanoma progression in human clinical specimens; (ii) overexpression of Sema 3A attenuates *in vitro* cell motility, invasiveness and proliferation in highly metastasic melanoma cells; (iii) tumor derived Sema 3A abrogated tumor-endothelial interaction via NRP1 mediated paracrine mechanism; (iv) Sema 3A promotes drug sensitivity of melanoma cells; (v) Sema 3A alone is not able to induce apoptosis however it enhances the apoptotic potential of curcumin and DTIC in B16F10 cells; (vi) overexpression of Sema 3A significantly diminished *in vivo* melanoma progression and angiogenesis in subcutaneous melanoma model, (vii) Sema 3A suppresses metastasis in melanoma models developed upon intracardiac injection and finally (viii) Sema 3A regulates melanoma cell migration and invasion through autocrine and paracrine mechanisms. Altogether, our experimental evidences suggested that Sema 3A acts as a potential tumor suppressor in melanoma model.

Angiogenesis has been demonstrated as one of the major event during malignant progression of cancer [47]. Recent advancement of cancer therapeutics indicated that targeting tumor angiogenesis could be one of the rational and promising approaches for the treatment of cancer. Earlier studies have shown that among various members of semaphorin family, Sema 3A could act as a potential suppressor of angiogenesis [13]–[16], [33], however the molecular mechanism underlying this process is not clearly understood and has been a field of intense investigation. In this study, using various *in vitro* as well as *in vivo* models, we have demonstrated that Sema 3A could suppress melanoma progression. Our clinical data for the first time revealed that melanoma growth negatively correlated with expression profile of Sema 3A. Earlier we have reported the crucial role of NRP1 in regulation of tumor endothelial interaction which ultimately regulates tumor angiogenesis [28]. In this study, we have shown that B16F10 overexpressed Sema 3A disrupts tumor-endothelial interaction through NRP1 mediated process. Furthermore, we have detected significant reduction of tumor angiogenesis in Sema 3A overexpressed clone when injected subcutaneously into C57BL/6 mice. In summary, our experimental observations have clearly indicated that Sema 3A may act as a potential suppressor of melanoma progression by attenuating angiogenesis.

Metastasis or the distant migration of cancer cells from the site of origin is the major cause of death by cancer [48]. Metastasis is a multistep process involving motility and invasion of cancer cells, intravasation, transit through vascular and/or lymphatic system, extravasation and growth of secondary tumor at new site [49]. Therefore, prevention of migration and metastasis of cancer cells is the center of attention for researchers and oncologists [48]. In this study, we have shown that Sema 3A attenuates *in vitro* melanoma cell motility and invasiveness. Moreover, our time lapse microscopy data have clearly indicated that Sema 3A significantly reduced the migration of melanoma cells. Earlier it has been reported that p53 inhibits lung metastasis in B16F10 cells [50]. We have also observed that overexpression of Sema 3A augmented the activation of p53 in various melanoma models. Moreover, we have correlated the Sema 3A and p53 phosphorylation at Ser-15 in melanoma clinical specimens. Therefore, the inhibitory effect of Sema 3A on melanoma cells may be p53 dependent, although extensive study is required to understand such mechanism. Furthermore, our allograft data have shown that Sema 3A overexpression drastically reduced *in vivo* melanoma growth and metastasis. Moreover, attenuation of tumor growth by intratumoral injection of CM of clone 2 indicates that tumor secreted Sema 3A also suppressed tumor growth via paracrine mechanism.

In recent time, treatment of cancer patients with anticancer agents/drugs (cancer chemotherapy) has shown greater promises; although there is some limitation of such therapy. Drug resistance of cancer cells has been known as the major burden for cancer chemotherapy and exhibit frequent clinical problem in patients. Therefore, development of novel therapeutic approach to overcome the drug resistance and increase the drug sensitivity of cancer cells remains a major challenge for the successful chemotherapy of cancer. In this study, we have noted that overexpression of Sema 3A in presence of various pharmacological anti-cancer agents (curcumin and DTIC) decreased cell survival as compared to control B16F10 cells. Moreover, we have observed that curcumin, even at comparatively lower doses significantly promotes apoptosis in Sema 3A overexpressed cells. Our live cell imaging data also suggested that fraction of control cells were escaped from apoptosis when they were incubated with curcumin (data not shown). Taken together, our experimental observations indicated that Sema 3A has no significant effect on melanoma cell survival but it increases the drug sensitivity of B16F10 cells.

This study highlights that Sema 3A attenuates the metastatic signature and angiogenic switch in melanoma model which ultimately suppresses melanoma progression. The data revealed that Sema 3A increases drug sensitivity of melanoma cells. The results demonstrate that chemotherapy of cancer by anti-cancer agents along with combination of Sema 3A could be a rational and promising approach for the treatment of cancer. The study suggests that Sema 3A regulated pathway may act as potentially important therapeutic target for the management of malignant melanoma.

## Supporting Information

Figure S1 **Expression profile of Sema 3A in human melanoma cells and its role in melanoma migration and invasion.** (**A**) Total RNA was isolated from human melanoma cells (A375 and SK-Mel-28) and Q-PCR analysis of Sema 3A expression was performed. Bar graph represents the normalized Sema 3A mRNA with GAPDH. *p<0.001. (**B**) Representative photographs of migrated/invaded melanoma cells were shown in S1B and the bar graphs were shown in Fig. 3B. (**C**) Invasion assays were performed with A375 and SK-Mel-28 cells either in absence or presence of Sema 3A recombinant protein. Invaded cell were counted and represented in the form of graph (Fig. S1C, panels I & II).
(TIF)

Figure S2 **Sema 3A inhibits melanoma migration and melanoma-endothelial cell interaction through paracrine mechanism.** (A) Representative photographs of migrated B16F10 cells showing Sema 3A abrogates melanoma migration through paracrine manner as described in Fig. 3C. (**B**) Photographs of migrated and invaded HUVEC showing Sema 3A attenuates melanoma-endothelial interaction as shown in Fig. 3D.
(TIF)

Figure S3 **Immunohistochemical analyses of human normal skin biopsy and malignant melanoma tissues by using anti-phospho-p53 antibody.** Two normal skin biopsy (**A**) and three malignant melanoma (**B**) specimens were analyzed immunohistochemically for visualizing the expression of Sema 3A (Fig. S3A, panels a, b & Fig. S3B panels a–c) and phospho-p53 of Ser-15 (Fig. S3A, panels e, f & Fig. S3B, panels g–i). Nuclei were stained with DAPI (Cy2 in green, Cy3 in red and DAPI in blue).
(TIF)

Figure S4 **Sema 3A controls melanoma cell proliferation.** SK-Mel-28 cells were plated on coverslip in 24 well plates. The cells were treated with 100 ng/ml Sema 3A for 24 h followed by incubation with complete media supplemented with BrdU for another 24 h. Cells were stained with BrdU labeling and detection kit, visualized under fluorescence microscope, counted and photographed (**A**) at 10× magnification and represented in the form of bar graph (**B**). *p = 0.0022. Nuclei were stained with DAPI.
(TIF)

Figure S5 **Time lapse videograph of wound migration of B16F10 cells.** Confluent monolayer of B16F10 cells was wounded and the migration of the cell towards the wound was monitored and photographed under Nikon time laps microscope in an interval of 10 min up to 18 h and represented in the form of video using Image Pro-Plus software after compiling the images into a movie.
(MP4)

Figure S6 **Time lapse videograph of wound migration of Sema 3A overexpressed B16F10 cells (clone 2).** Confluent monolayer of clone 2 cells was wounded and the migration of the cell towards the wound was monitored and photographed under Nikon time laps microscope in an interval of 10 min up to 18 h and represented in the form of video using Image Pro-Plus software after compiling the images into a movie.
(MP4)

Figure S7 **Time lapse videograph of wound migration of B16F10 cells treated with conditioned media (CM) of Sema 3A overexpressed B16F10 cells (clone 2).** Confluent monolayer of control cells was wounded, treated with CM collected from clone 2 cells and the migration of cells toward the wound was monitored and photographed under Nikon time laps microscope in an interval of 10 min up to 18 h and represented in the form of video using Image Pro-Plus software after compiling the images into a movie.
(MP4)
