# Supplementary figures and images for: Semaphorin 3A Suppresses Tumor Growth and Metastasis in Mice Melanoma Model
Source: PLoS One. 2012 Mar 20;7(3):e33633. doi: 10.1371/journal.pone.0033633 (PMC3308985; doi:10.1371/journal.pone.0033633)

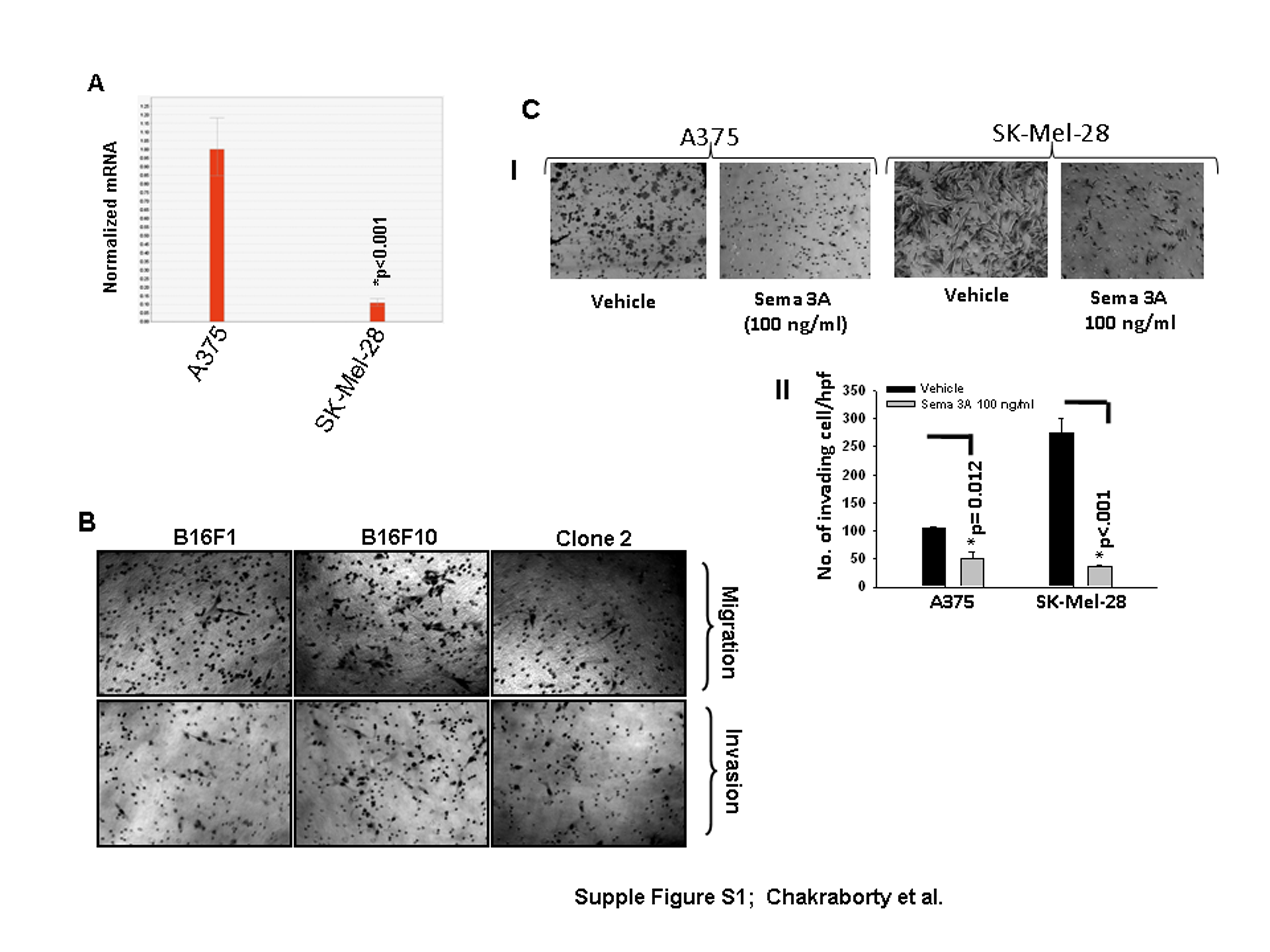

Supplement: Figure S1 — Expression profile of Sema 3A in human melanoma cells and its role in melanoma migration and invasion. (A) Total RNA was isolated from human melanoma cells (A375 and SK-Mel-28) and Q-PCR analysis of Sema 3A expression was performed. Bar graph represents the normalized Sema 3A mRNA with GAPDH. *p<0.001. (B) Representative photographs of migrated/invaded melanoma cells were shown in S1B and the bar graphs were shown in Fig. 3B. (C) Invasion assays were performed with A375 and SK-Mel-28 cells either in absence or presence of Sema 3A recombinant protein. Invaded cell were counted and represented in the form of graph (Fig. S1C, panels I & II). (TIF) [file pone.0033633.s001.tif]

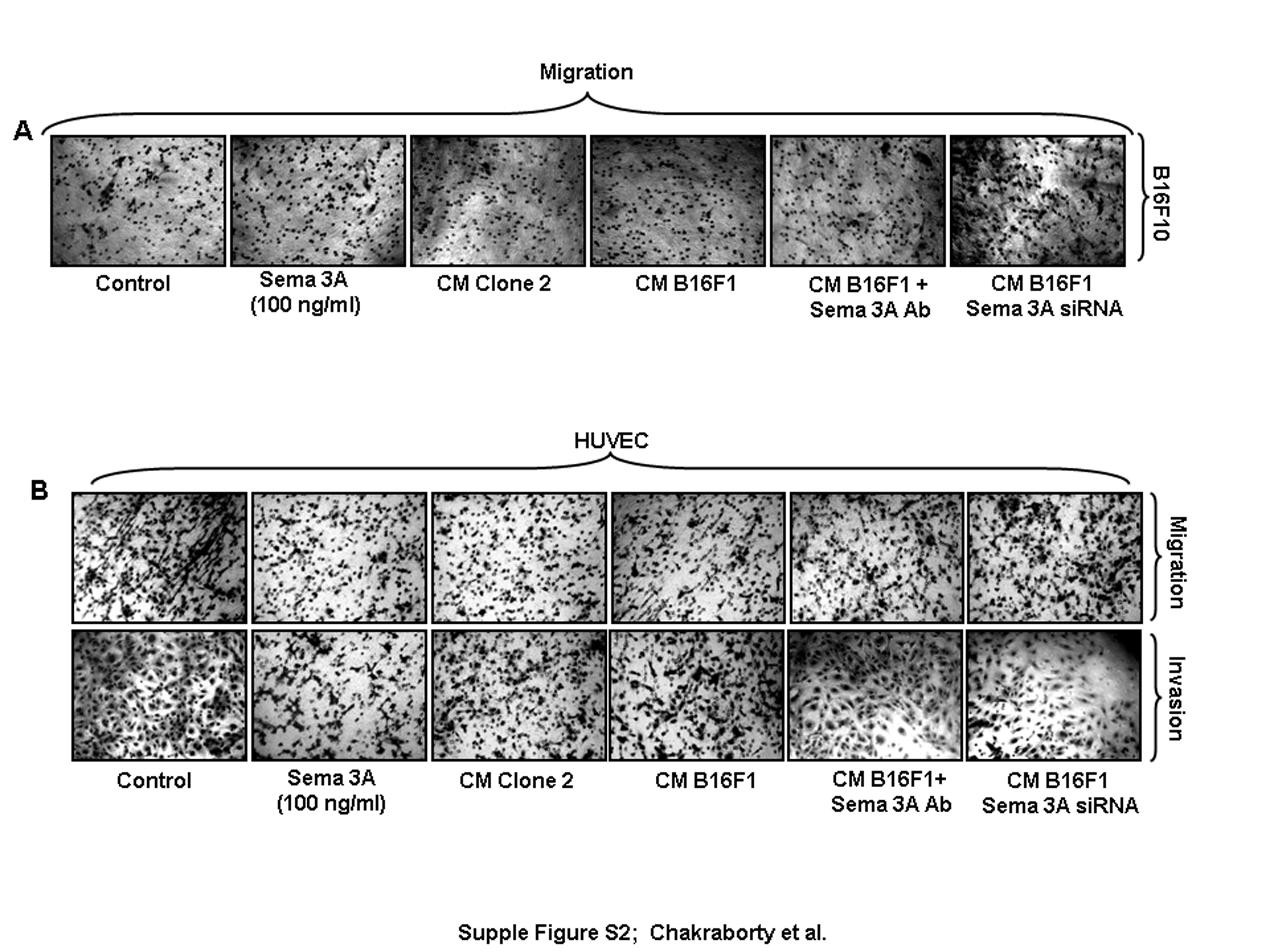

Supplement: Figure S2 — Sema 3A inhibits melanoma migration and melanoma-endothelial cell interaction through paracrine mechanism. (A) Representative photographs of migrated B16F10 cells showing Sema 3A abrogates melanoma migration through paracrine manner as described in Fig. 3C. (B) Photographs of migrated and invaded HUVEC showing Sema 3A attenuates melanoma-endothelial interaction as shown in Fig. 3D. (TIF) [file pone.0033633.s002.tif]

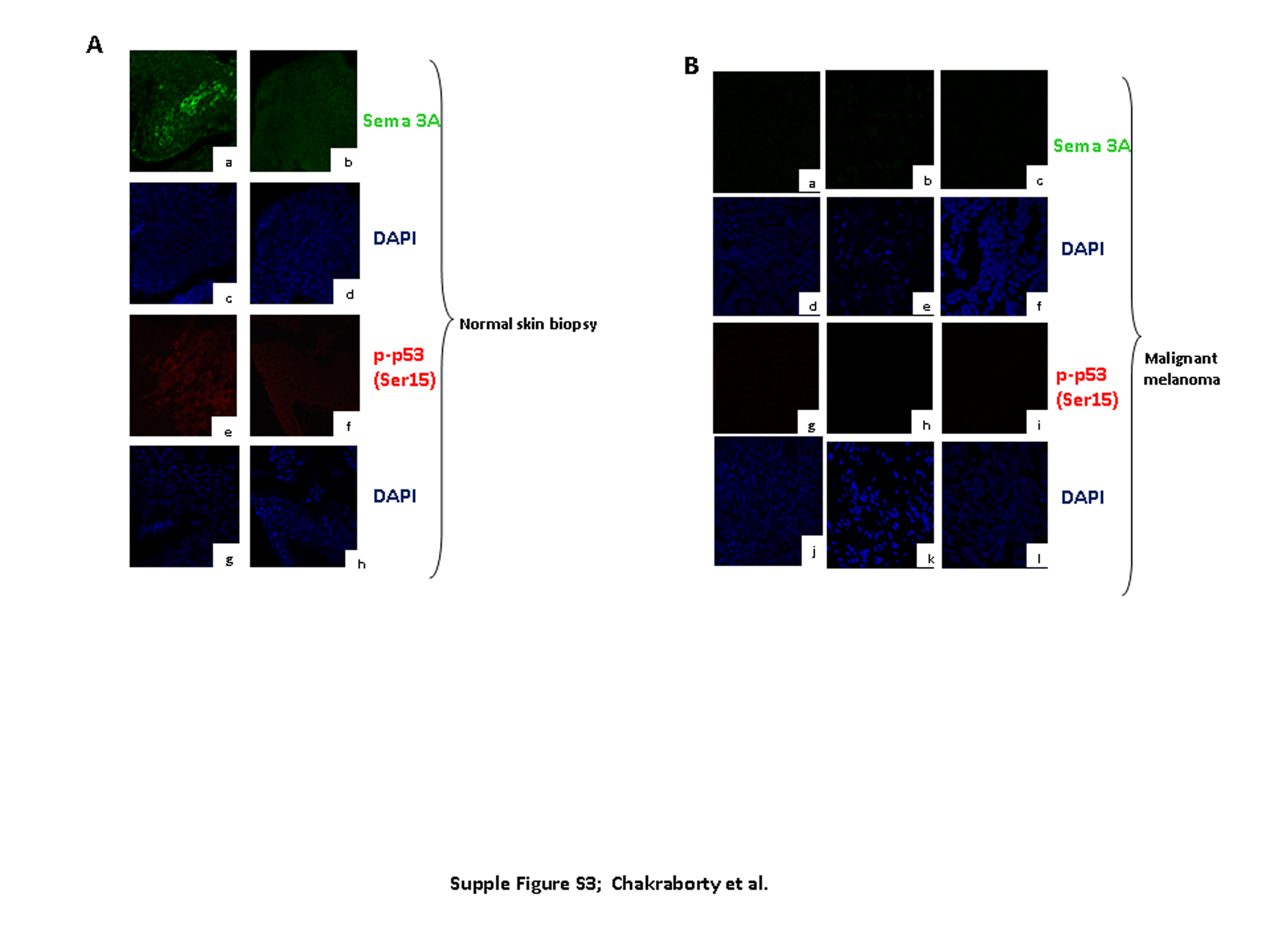

Supplement: Figure S3 — Immunohistochemical analyses of human normal skin biopsy and malignant melanoma tissues by using anti-phospho-p53 antibody. Two normal skin biopsy (A) and three malignant melanoma (B) specimens were analyzed immunohistochemically for visualizing the expression of Sema 3A (Fig. S3A, panels a, b & Fig. S3B panels a–c) and phospho-p53 of Ser-15 (Fig. S3A, panels e, f & Fig. S3B, panels g–i). Nuclei were stained with DAPI (Cy2 in green, Cy3 in red and DAPI in blue). (TIF) [file pone.0033633.s003.tif]

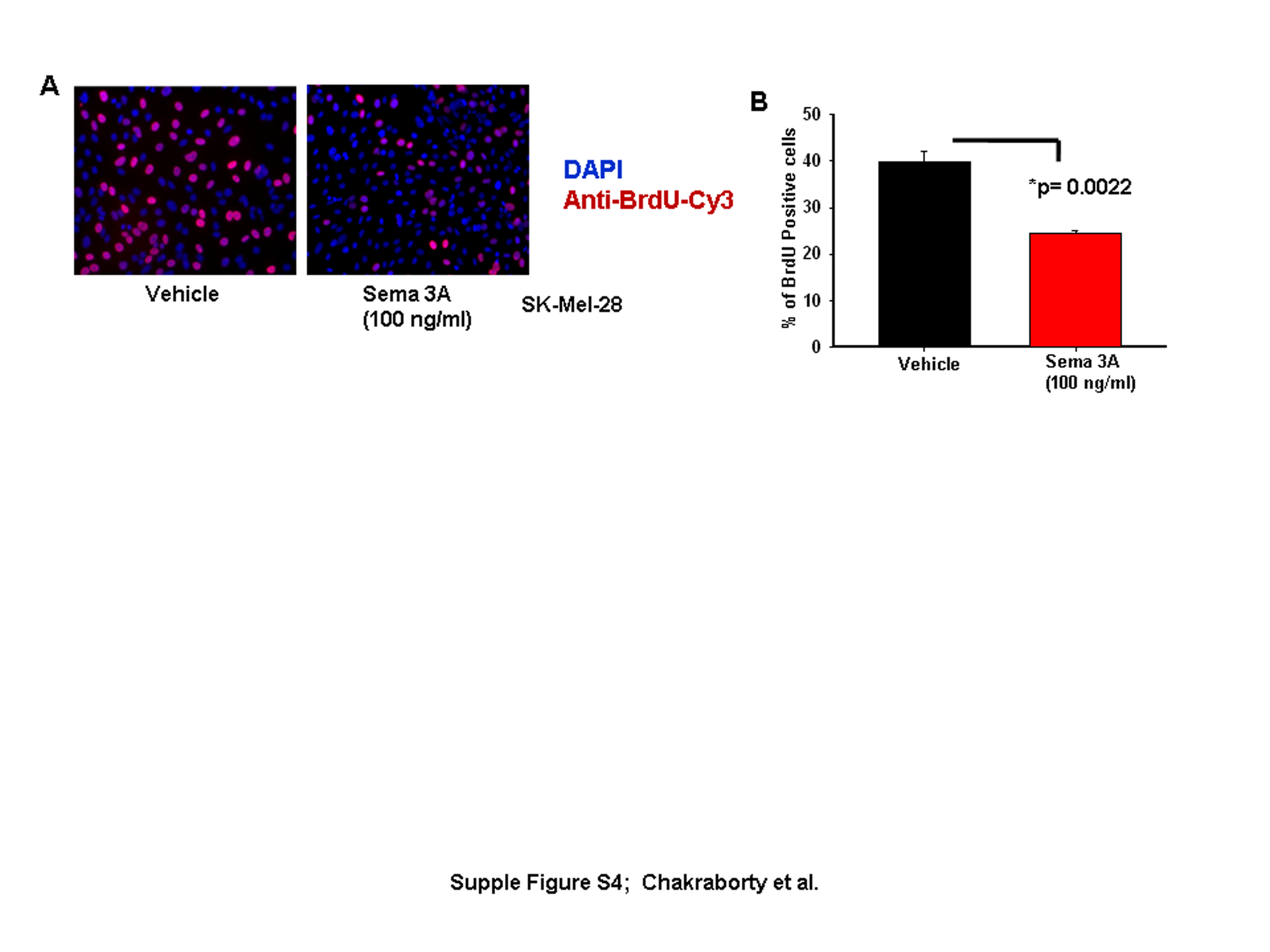

Supplement: Figure S4 — Sema 3A controls melanoma cell proliferation. SK-Mel-28 cells were plated on coverslip in 24 well plates. The cells were treated with 100 ng/ml Sema 3A for 24 h followed by incubation with complete media supplemented with BrdU for another 24 h. Cells were stained with BrdU labeling and detection kit, visualized under fluorescence microscope, counted and photographed (A) at 10× magnification and represented in the form of bar graph (B). *p = 0.0022. Nuclei were stained with DAPI. (TIF) [file pone.0033633.s004.tif]
